# The impact of digital literacy on rural residents' sports participation behavior:evidence from China

**DOI:** 10.3389/fpubh.2026.1781770

**Published:** 2026-04-09

**Authors:** Jiawei Chen, Meng Xiang, Qifei Xia

**Affiliations:** 1 Department of General Education, Hunan Mechanical & Electrical Polytechnic, Changsha, Hunan, China; 2 School of Physical Education and Arts, Hunan University of Medicine, Huaihua, Hunan, China; 3 School of Physical Education, Ankang College, Ankan, Shaanxi, China

**Keywords:** digital literacy, digital usage behaviour, human capital accumulation, level of social capital, sports participation behavior

## Abstract

**Purpose:**

Insufficient Digital Literacy among residents may lead to a polarization in access to digital resources, thereby affecting their willingness to participate in exercise, which necessitates attention. This study aims to analyze the impact of Digital Literacy on sports participation behavior from the perspective of internet perception, along with its potential mechanisms, providing a basis for promoting healthy lifestyles and enhancing public health service levels in rural areas.

**Methods:**

Based on the mixed cross-sectional survey data from the China family panel studies (CFPS) conducted in 2018, 2020, and 2022, a comprehensive analysis was performed using binary logistic regression, Heckman two-stage regression, instrumental variable methods, and Karson-Holm-Breen (KHB) mediation effects to examine the impact of Digital Literacy on sports participation behavior among rural residents and its underlying mechanisms.

**Results:**

This study found that Digital Literacy significantly enhances the level of sports participation behavior among rural residents, with results remaining reliable after a series of robustness checks. Furthermore, the mechanism analysis indicates that Digital Literacy can indirectly enhance the level of sports participation behavior by stimulating digital usage behaviors among rural residents, promoting human capital accumulation, and increasing social capital levels. According to the heterogeneity analysis results, male, middle-aged, and married rural residents exhibit higher levels of Digital Literacy and are more inclined to participate in sports activities.

**Conclusion:**

As the Digital Literacy level of rural residents improves, a deeper understanding of the multidimensional value of physical exercise is attained, thereby promoting their active participation. Mechanisms related to digital usage behavior, human capital accumulation, and social capital levels play a more significant role in facilitating physical exercise. To accelerate the construction of digital villages, it is imperative to increase investments in rural digital education to address the challenges faced by residents in utilizing internet technology and devices, thereby facilitating better integration into an information society and fulfilling the demands of rural communities for physical exercise and healthy living.

## 1 Introduction

Against the macro background of the in-depth development of China's Digital Rural Strategy and its integration with the National Fitness Strategy, digital technologies have been deeply embedded in the core areas of public service provision for rural sports and the dissemination of fitness information, driving a qualitative transformation in the channels and practice models of sports participation among rural residents. However, the heterogeneous distribution of digital literacy levels among rural residents has emerged as a key limiting factor in their effective participation in sports activities through digital media. According to the General Administration of Sport of China's “2025 National Fitness Participation Survey Report,” the proportion of rural residents who frequently engage in physical exercise is only 31.1% in 2025, which is 11.8 percentage points lower than that of urban residents (1); The Cyberspace Administration of China's ‘'Report on the Development Level of National Digital Literacy and Skills (2024)” indicates that only 50.57% of rural adults possess basic digital literacy and skills, significantly lower than the 65.92% of urban residents (2). Research in the field of sports indicates that the proportion of rural residents who regularly participate in physical exercise has long been lower than that of urban residents, and insufficient ability to use digital tools has become an important limiting factor (3). Therefore, it is necessary to gain an in-depth understanding of the intrinsic mechanisms and boundary conditions between digital literacy and the sports participation levels of rural residents.

Limited studies have analyzed the relationship between the two from the perspective of internet perception. Research on digital literacy and sports participation has primarily focused on the mechanisms or contexts through which digital technology affects sports engagement (4), investigating the antecedents of sports participation among rural residents (5) and analyzing the outcomes of digital literacy on health-related behaviors (3). The existing findings provide substantial theoretical support for this study. In this context, the study utilizes data from the China Family Panel Studies (CFPS) conducted in 2018, 2020, and 2022 to explore the “black box” of how digital literacy influences sports participation among rural residents. This work aims to enrich the understanding of the mechanisms linking digital technology and health behaviors, with a focus on the weak sports development context in rural areas. It intends to provide precise empirical evidence and policy references for the deep integration of the digital rural strategy and the national strategy for fitness promotion.

## 2 Literature review and research hypotheses

### 2.1 Digital literacy and rural residents' sports participation behavior

The “Action Outline for Enhancing National Digital Literacy and Skills (2022–2035),” issued by the Central Cybersecurity and Informatization Committee of China in 2021, incorporates national digital literacy and skills into the strategic framework of China, defining it as a collection of qualities and competencies, including digital acquisition, creation, usage, evaluation, interaction, sharing, innovation, security assurance, and ethics that citizens in a digital society should possess for their learning, work, and daily life. Building on existing research (6, 7), this study defines the digital literacy affecting the sports participation of rural residents as a comprehensive digital competency system that encompasses the entire process of sports engagement—including decision-making, implementation, and sustained involvement—necessary for achieving effective and high-quality participation. This definition echoes the core logic of the Technology Acceptance Model proposed by Davis based on the Theory of Reasoned Action (8). The model provides a fundamental framework for analyzing the internet perception of rural residents, asserting that in any context, a rational individual's perceptions of the internet are determined by their perceived usefulness, which is influenced by factors such as ease of use and policy support. The recognition among rural residents of the importance of digital technology and its application in contexts such as sports participation forms the logical starting point for the empowerment of sports participation through digital literacy; this perspective not only transcends the general definition of digital literacy but also aligns better with the realities of resource scarcity and limited digital application scenarios in rural sports. It thus establishes a conceptual and theoretical foundation for subsequent empirical measurement and mechanism analysis. Existing literature has conducted valuable explorations around the promotion of healthy behaviors through digital literacy. A survey study has indicated that rural residents with higher digital literacy are more inclined to explore various digital health interventions (such as physical exercise and online health consultations) to enhance their well-being (9). When residents utilize digital tools or devices correctly, they can better share and interact with their fitness peers, exchange health resource information, and increase the frequency of joint exercise activities (10). Furthermore, when elderly individuals perceive that digital technologies, such as mobile applications, smart wearable devices, and chat AIs, are beneficial for enhancing self-management of health, they tend to make efforts to learn and master these technologies, resulting in improved online health behaviors. Li et al. conducted a longitudinal study using panel data from the CFPS (2018–2022) and found that digital literacy has a long-term stable positive driving effect on both the participation rate and frequency of physical fitness among residents (11). By integrating the contextual constraints of rural sports participation with the aforementioned multidimensional empirical evidence, the enhancement of digital literacy oriented toward the process of sports participation can effectively address the informational barriers, resource limitations, and technological obstacles faced by rural residents, thereby promoting a comprehensive increase in their participation frequency, duration, and quality. Based on this, the present study proposes the following hypothesis:

H1: Digital Literacy has a positive impact on the sports participation behavior of rural residents.

### 2.2 The mechanism of digital literacy's influence on rural residents' sports participation behavior

Despite the potential close relationship between rural residents' Digital Literacy and sports participation behavior, the underlying pathways of transmission remain unclear. The influencing pathways of Digital Literacy are extensive and diverse, encompassing multiple direct and indirect mechanisms, such as human capital and digital usage behaviors, which sustain residents' active participation in sports activities. Therefore, this study posits that Digital Literacy influences rural residents' sports participation behavior through three channels: incentivizing digital usage behaviors, promoting human capital accumulation, and enhancing social capital levels.

First, the digital usage behaviors. Individual Digital Literacy is inextricably interconnected with digital usage behaviors. Digital usage behaviors encompass contemporary technological practices, such as browsing online information, interacting on user platforms, engaging in recreational activities, and expressing daily experiences, thereby enriching the content and forms of digital life. The Comprehensive Model of Information Seeking (CMIS) indicates that health-related factors can influence health information-seeking behaviors through the utility of relevant carriers (12). Existing research has confirmed that individuals who place greater emphasis on information regarding diet, weight, or physical activity, coupled with higher accessibility to technological devices, are more likely to engage in health-promoting behaviors (13). For rural residents, individuals possessing Digital Literacy and skills can better leverage social networking platforms to share, communicate, and disseminate information related to sports, thereby expressing their concerns about fitness online (14). For instance, utilizing platforms such as Keep, Fit Time, and Nike + Training Club to post images and videos enables users to receive timely guidance and assistance from professionals, achieving scientific and efficient workouts (15). Moreover, rural residents have limited access to digital devices, often restricted to operating a singular interface, resulting in relatively limited functionality (16). Enhancing Digital Literacy not only allows them to transition from simple information engine searches to online interactions and social learning (17), but also enables the use of short videos and platforms such as Facebook for fitness content creation, achieving dual development in profit generation and health promotion (18).

Second, the accumulation of human capital. Human capital is defined as the knowledge, skills, abilities, and experiences possessed by individuals. At present, the application of digital technology is complex and abstract. Although residents receive appropriate prompts and assistance during operations, the limited capabilities and understanding of rural residents, coupled with a lack of systematic knowledge and skills training, may hinder their ability to fully enjoy the “digital dividend.” Bandura's social learning theory emphasizes the dynamic process through which individuals acquire knowledge by gathering behavioral cues from their social environment and subsequently enhance relevant behavioral competencies through digestion, absorption, and innovation (19). The improvement of digital literacy among rural residents exemplifies a typical dynamic process of social learning, in which individuals learn to identify online interfaces, establish personal security measures, and integrate into the digital participation environment through a series of processes including independent exploration, seeking advice humbly, and practical application. This process thereby enhances their adaptability and enables them to actively benefit from digital technologies (20). Research has found that digital literacy enables rural residents to efficiently and accurately access relevant digital learning resources and fill knowledge gaps, thereby enhancing experiential learning of professional knowledge and skills and improving overall competencies (21). Furthermore, the enhancement of human capital signifies that residents can participate in collective physical exercise through social media and online communities (22). For example, rural residents can utilize digital technologies to filter and select irrelevant health resource information (23), accurately seeking more online communities of interest, thus providing opportunities for promoting the occurrence and sustainability of healthy behaviors. It is noteworthy that, prior to this, browsing the internet for health information tailored to their needs was a complex and daunting task for most rural residents (24). We hypothesize that the accumulation of human capital may serve as an important transmission pathway.

Third, social capital levels. Social capital is defined as a collection of actual or potential resources, fundamentally based on the resources formed through individual social relationships (25). Compared to traditional media, the new generation of digital technologies is shaping a completely new lifestyle, characterized by richer and more diverse content and forms. Rural China emphasizes the intertwining of kinship, geographical relationships, and various expanding social connections (26). Rural residents with high Digital Literacy are more adept at overcoming technological application barriers, skillfully utilizing digital tools such as smartphones and computers to expand their social networks, establish interactive platforms, and maintain sharing and communication, thereby creating a favorable environment for sports learning, competition, and exchange. For instance, a study conducted in South Korea indicates that social capital serves as a transmission pathway between Digital Literacy and the life satisfaction of older adults, particularly playing an important bridging role in maintaining and expanding relationships with family, friends, and the community (27). Additionally, there exists a significant correlation between social capital and sports participation behavior. Previous studies have identified that the scale of personal relationship networks, frequency of interaction, and homogeneity of network members are crucial for promoting residents' participation in sports (28). Research conducted by Pan et al., based on a large-scale survey in China, reveals that digital lifestyles have broadened the “circle of friends” horizontally, breaking spatial limitations of social interactions and enhancing residents' willingness to engage in physical exercise (29). Furthermore, research from the Chicago Community Survey found that neighborly trust and norms of reciprocity significantly impact the daily exercise behaviors of community residents (30). Similar findings have been reported in studies conducted in Japan, where older adults involved in sports community organizations are less likely to stay home and exhibit greater interest in participating in recreational activities such as baseball and running (31). Although some studies have investigated the mechanisms through which social capital operates in various domains, research focusing on the social capital levels of rural residents remains insufficient. In light of these considerations, this study proposes the following hypotheses based on the aforementioned review:

H2a: Digital Literacy promotes sports participation behavior by motivating rural residents' digital usage behaviors.

H2b: Digital Literacy enhances sports participation behavior by facilitating the accumulation of human capital among rural residents.

H2c: Digital Literacy promotes sports participation behavior by enhancing the level of social capital among rural residents.

**Figure 1:**
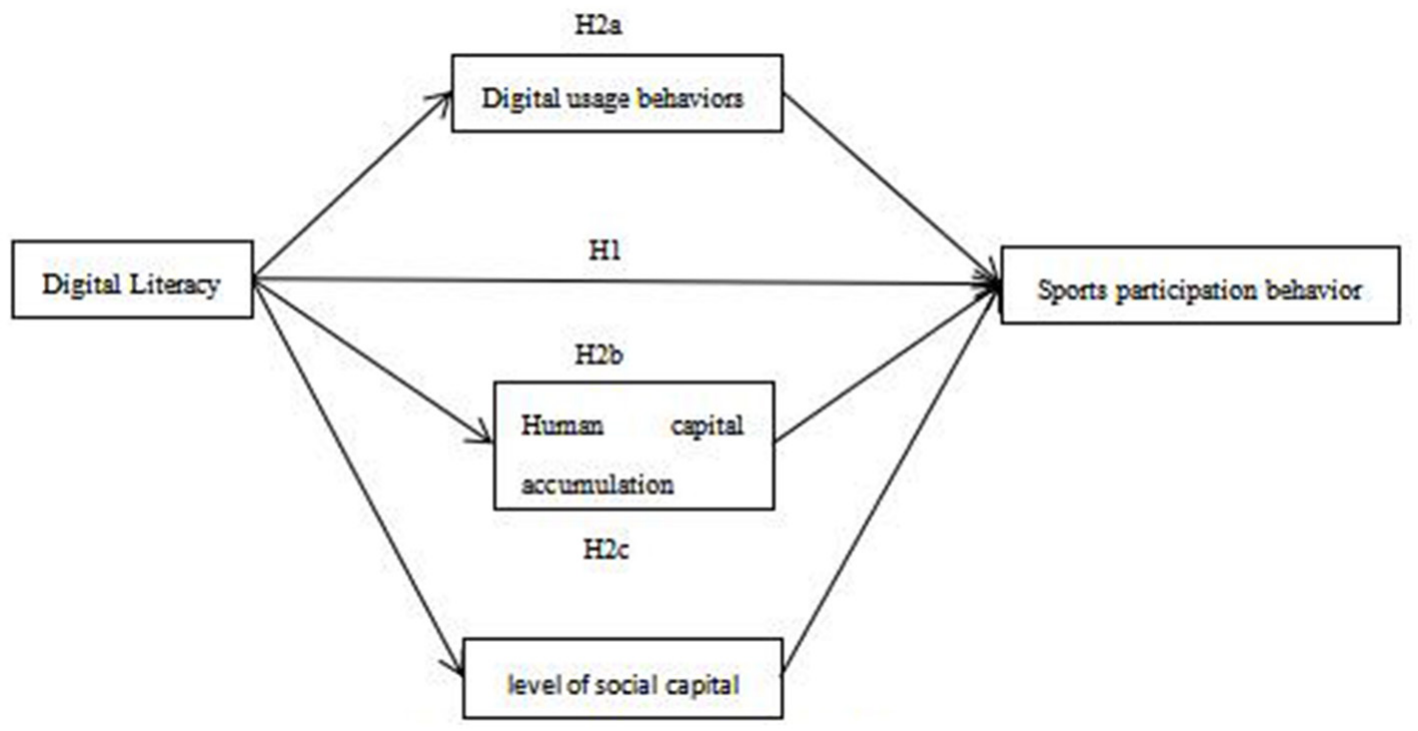
Theoretical framework model.

Based on the literature and hypotheses outlined above, this study constructs a framework model illustrating the impact of Digital Literacy on sports participation behavior among rural residents. See Figure 1.

## 3 Data sources and research design

### 3.1 Data sources

The research data is sourced from the China Family Panel Studies (CFPS, 2020) conducted by the Institute of Social Science Survey (ISSS). A population-proportional sampling method was employed to conduct household surveys across all age groups in 31 provincial-level administrative regions of China. This questionnaire includes questions related to residents' internet usage, which reflect their level of digital literacy. Regarding the measurement of sports participation behavior, this questionnaire provides detailed data support for the research through various aspects such as exercise duration and frequency. Currently, the CFPS data has been updated to the most recent year of 2022. We selected data from the years 2018, 2020, and 2022, merging them into a mixed cross-sectional dataset. After selecting relevant research variables and excluding samples with missing values and invalid responses (including “refusal to answer”), a total of 19,815 valid samples from rural residents were obtained.

#### 3.1.1 Data processing and handling of missing values

Data processing: this study utilized the CFPS datasets from 2018, 2020, and 2022 to construct a combined cross-sectional dataset, with the merging process strictly adhering to the official data usage guidelines of CFPS. First, a baseline for sample matching was established. The unique identification code (ID) of respondents was used as the primary matching key, ensuring that the observations of the same respondent in different years could be traced, while samples with duplicated or invalid IDs across the years were excluded. Second, the sample scope was defined. Only the observations of respondents explicitly marked with a rural household registration in the three datasets were retained, while samples with urban household registration, missing household information, or unclear details were excluded, ultimately forming an initial pool of 28,642 rural residents. Finally, data cleaning was performed, with specific procedures as follows: First, the treatment of outliers was conducted. For continuous variables, the ±3 standard deviations rule was employed to identify outliers, and samples characterized by an age ≤ 16 years (not reaching the legal adult standard), a social capital level of 0 without reasonable answering basis, were excluded. Second, the handling of logical inconsistencies was performed. Samples exhibiting logical inconsistencies among the core variables were classified as invalid and excluded. Third, the handling of invalid responses was carried out. Samples that selected “refused to answer,” “don't know,” or “no answer” for core variables in the questionnaire were excluded, ensuring that only valid response observations were retained. Ultimately, 21,568 valid samples remained.

Handling of missing values: this study employs multiple imputation (MI) to address missing values. Firstly, an analysis of the status of missing values was conducted. After data cleaning, a remaining sample of 21,568 observations was obtained, with the missing rate of core variables ranging from 1.2 to 5.8%, satisfying the applicability criteria of Multiple Imputation. Secondly, the imputation model was established. With the core variables as the target variables, control variables and survey year dummy variables were included as auxiliary variables to construct a multinomial logistic imputation model and a linear imputation model. Subsequently, the imputation process was executed and verified. The imputed dataset generated using the mi impute command in Stata 17.0 was consolidated according to Rubin's combining rules; validation revealed that the means and standard deviations of the imputed variables showed no significant differences from the original valid sample (p > 0.05), indicating that the imputation results are reliable. Finally, the final sample was determined. After dealing with all missing data, a final valid sample of 19,815 observations was obtained.

### 3.2 Variable selection

#### 3.2.1 Dependent variable

The dependent variable is the sports participation behavior of rural residents. According to existing research (32–34), regular participation in physical exercise is a planned, structured, and repetitive sociocultural activity, with the frequency and duration of exercise typically chosen to reflect the physical activity levels of the population. In the CFPS data, the types of physical activities are diverse and varied, typically including periodic exercises such as walking, long-distance running, jogging, and hiking; ball games like football, basketball, and table tennis; as well as water sports such as swimming, sailing, and diving. Relevant studies indicate (35) that the total amount of individual physical activity needs to be assessed from multiple aspects, including exercise frequency, duration, and intensity. The American College of Sports Medicine (ACSM) recommends that individuals engage in moderate physical activity at least three times a week, with each session lasting 30 min or longer (36). The CFPS primarily reflects whether residents regularly participate in physical exercise through two questions: (1) frequency of exercise. The questionnaire includes the question, ‘'How often do you participate in sports and leisure activities?” Responses such as ‘'never, less than once a month, more than once a month, once or twice a week, three to four times a week, five or more times a week, once a day, and twice or more a day” are assigned values from 1 to 8. Based on ACSM recommendations, responses indicating ‘'an average weekly exercise frequency of ≥3–4 times” and above are defined as participating in physical exercise, assigned a value of 1; otherwise, a value of 0 is assigned. (2) Duration of exercise. The questionnaire also includes the item, ‘'How many minutes do you exercise each time, on average?” Each instance of exercise lasting 30 min or more is assigned a value of 1; otherwise, a value of 0 is assigned. Participation in physical exercise is defined as having responses of ‘'an average weekly exercise frequency of ≥3 times and a duration of ≥30 min per session,” which is assigned a value of 1; otherwise, a value of 0 is assigned.

#### 3.2.2 Independent variable

The independent variable is the Digital Literacy of rural residents. Previous research has shown that many scholars assess Digital Literacy using a single dimension, primarily evaluating whether individuals use the internet, which has certain limitations. Drawing on the research of Wang et al (37), we will reflect individuals' Digital Literacy levels through their understanding of the functional aspects of internet technology. This includes the following dimensions: digital work literacy (the importance of the internet for work), digital entertainment literacy (the importance of the internet for leisure and entertainment), digital social literacy (the importance of the internet for maintaining contact with family and friends), digital learning literacy (the importance of the internet for learning), and digital life literacy (the importance of the internet for daily life). Each dimension will be classified into five levels of importance, assigned scores ranging from 1 to 5 in ascending order of ‘'not important at all” to ‘'very important.” Factor analysis was employed to reduce the dimensionality of the five dimensions, resulting in the extraction of one common factor, referred to as the Digital Literacy common factor. The cumulative variance contribution rate of Digital Literacy is 66.4%, with factor loadings ranging from 0.491 to 0.648. The KMO measure is 0.788, and Cronbach's α is 0.727, indicating a satisfactory level of correlation among the dimensions. In summary, higher values indicate a greater level of Digital Literacy among individuals.

#### 3.2.3 Mediator

The mediating variables encompass three dimensions: digital usage behavior, human capital accumulation, and social capital levels. (1) Digital usage behavior. Digital technologies include devices such as computers, mobile phones, and tablets. Specifically, if rural residents can utilize mobile internet or PC devices, they possess a high level of digital literacy, enabling them to understand and access information resources. Referring to existing studies (38), digital usage behavior among residents is defined by two aspects: whether they access the internet via mobile devices or computers. If this behavior is present, it is assigned a value of 1; otherwise, it is assigned a value of 0. (2) Human Capital Accumulation. The human capital theory proposed by American economist Schultz posits that the level of knowledge and skills possessed by workers is a key factor in promoting human capital accumulation (39). The CFPS data includes two aspects: whether individuals engage in learning and the frequency of using the internet for learning. Human capital is calculated by averaging these variables; if the level of human capital accumulation is high, it is assigned a value of 1; otherwise, it is assigned a value of 0, making it a binary variable. (3) Social Capital Levels. According to Coleman's perspective, trust, as a form of social relationship, constitutes an important aspect of social capital (40). Furthermore, social capital is defined as social resources embedded within interpersonal networks, and interpersonal interactions play a significant role in social capital (41). Therefore, based on the actual situation of the survey, we selected three dimensions from the CFPS data—parental trust, neighbor trust, and interpersonal relationships—to construct an individual's social capital level, which ranges from 0 to 10; a higher value indicates a greater level of social capital.

#### 3.2.4 Control variable

In the present study, control variables were selected by following approaches adopted in existing literature (42–44), which mainly include the following aspects: Respondents' gender (female = 0, male = 1); Age (calculated as the survey year minus the year of birth); Educational level (0 = primary school or below, 1 = junior high school, 2 = senior high school (general senior high school, secondary vocational school, technical school), 3 = junior college, 4 = bachelor's degree or above); Political status (non-Party member = 0, Communist Party of China (CPC) member = 1); Marital status (unmarried = 0, married = 1); Health status (1 = unhealthy, 2 = average, 3 = relatively healthy, 4 = healthy, 5 = very healthy). Descriptive statistics of all variables are presented in Table 1.

**Table 1:** Descriptive statistics for each variable.

Variable	Assignment Instructions	Mean	Std.	min	max
Dependent variable					
Sports participation behavior	Exercise frequency (≥3 times) + Exercise duration (over 30 min per session)	0.513	0.500	0	1
Independent variable					
15.6-7.4,-25.7499ptDigital literacy	Factor analysis was performed on the importance of the Internet perceived by rural residents in learning, daily life, work, and other aspects, and the comprehensive factor score was calculated.	3.926	0.783	1	5
Mediating variable					
Digital usage behavior	Whether using mobile Internet and whether using computer Internet were summed to calculate the value, where “No” = 0 and “Yes” = 1.	0.355	0.479	0	1
Human capital accumulation	Whether engaging in reading and whether engaging in online learning were summed to calculate the composite value, where “No” = 0 and “Yes” = 1.	0.355	0.380	0	1
Level of social capital	Parental trust, neighborhood trust, and interpersonal relationship were summed and calculated; the higher the resulting value, the higher the level of social capital.	7.694	1.129	0	10
Control variables					
Gender	Female = 0, Male = 1	0.497	0.500	0	1
Age	Actual age	39.33	12.822	18	100
Educational level	0 = Primary school and below 1 = Secondary school 2 = Senior secondary education (comprehensive secondary school, technical school, specialized secondary school) 3 = College diploma 4 = Bachelor's degree and above	1.767	1.546	0	4
Political affiliation	0 = General public, 1 = Member of the Communist Party of China	0.020	0.139	0	1
Marital status	0 = Unmarried, 1 = Married	0.727	0.445	0	1
Health status	1 = Unhealthy, 2 = Average, 3 = Fairly healthy, 4 = Very healthy, 5 = Extremely healthy	2.726	1.055	1	5

### 3.3 Model construction

#### 3.3.1 Benchmark regression model

In this study, all data analyses were conducted using Stata 17.0 for statistical processing. As the dependent variable, participation in physical exercise, is a binary variable, the direct application of the ordinary least squares (OLS) method may lead to estimation bias; conversely, the binary Logit model effectively examines the relationship between the binary response dependent variable and a set of explanatory variables (influencing factors). Therefore, a binary Logistic model was constructed to estimate the impact relationship between digital literacy and participation in physical exercise among rural residents. The model's specification is based on the classical theoretical framework of discrete choice models (45), following the application norms established by Wooldridge (46) for research on binary behavior decision-making. The specific model specification is as follows:


$$\begin{array}{l} In\left(\frac{P}{1-P}\right)=\alpha +\beta DL+\chi_{i}\gamma_{i} \end{array}$$ (1)

In Equation (1), *DL* represents the level of digital literacy, *P* denotes the participation of rural residents in physical exercise, β signifies the impact coefficient of digital literacy on the participation of rural residents in physical exercise, *X*_*i*_ indicates the control variables, and γ_*i*_ represents the effect coefficients of the control variables on the participation of rural residents in physical exercise. To mitigate the issue of sample selection bias, this study employs the Heckman model. The core principle of this model and the two-stage estimation logic follows the basic framework of the sample selection model proposed by Heckman (47). The specific application adheres to the empirical research standards set forth by Angrist & Pischke (48). The fundamental approach is as follows: First, a correction factor, the inverse Mills ratio λ, is constructed after estimating the level of digital literacy among rural residents in the first stage; second, λ is incorporated as a control variable into the regression model; this allows for testing the effect of λ to determine whether the self-selection of digital literacy among rural residents influences the results. The specific model specification is as follows:


$$\begin{array}{l} \lambda =\varphi \left(\beta_{0}+\overset{n}{\underset{i=1}{\sum }}\beta_{i}X_{i}\right)÷\left[1-\phi \left(\beta_{0}+\overset{n}{\underset{i=1}{\sum }}\beta_{i}X_{i}\right)\right] \end{array}$$ (2)

In Equation (2), ϕ *(*•*)* represents the probability density function of the standard normal distribution, while ø* (*•*)* denotes the cumulative probability distribution function of the response.

#### 3.3.2 Mediating effect model

To verify the existence of mediation effects, four approaches were employed in this study to assess the mediating role. First, a stepwise testing method was used to construct the mediation effect model, preliminarily verifying the existence of mediation effects. This testing framework is established according to the classic criteria of Baron & Kenny (49), and the equations for estimating the mediation effect model are as follows:


$$\begin{array}{l} Exercise=\alpha_{0}+\alpha_{1}DL+\alpha_{x}control+\delta_{i} \end{array}$$ (3)

$$\begin{array}{l} \text{Intermediary variables}=\beta_{0}+\beta_{1}Exercise+\beta_{x}control+\delta_{i} \end{array}$$ (4)

$$\begin{array}{l} Exercise=\gamma_{0}+\gamma_{1}DL+\gamma_{2}Ir+\gamma_{3}Control+\delta_{i} \end{array}$$ (5)

In Equations (3–5), Exercise represents participation in physical exercise, *DL* denotes the digital literacy level of rural residents, *IV* indicates the mediating variable digital usage behavior, and human capital accumulation and social capital levels are included as additional variables, while *Control* refers to a series of control variables and δ represents a random disturbance term. In equation (5), γ_1_ and γ_2_ are the coefficients representing the effects of digital literacy and the mediating variable on the participation of rural residents in physical exercise after incorporating control variables, with γ_1_ indicating the direct effect. If the mediation effect is significant, it is required that α_1_, β_1_, and γ_2_ exhibit significance. At this point, if γ_1_ is not significant, a full mediation effect is indicated. If γ_1_ is significant and γ_1_ < α_1_, a partial mediation effect is identified. Furthermore, traditional mediation analysis methods have often conflated confounding effects and scale effects, while the KHB model addresses this issue; therefore, the KHB method was also employed for effect decomposition. Finally, to enhance the reliability of the conclusions, both the Sobel test and the Bootstrap test were employed to further verify the existence of the mediation effect.

## 4 Empirical results

### 4.1 Baseline regression

Table 2 presents the estimation results of the impact of Digital Literacy on rural residents‘ sports participation behavior. Among them, Regression (1) only considers the univariate relationship between Digital Literacy and rural residents' sports participation behavior. It was found that Digital Literacy exerts a significantly positive impact on rural residents‘ sports participation behavior (β = 0.289 < 0.001), indicating that for each one-unit increase in rural residents' Digital Literacy, the probability of participating in physical exercise increases by 28.9%. Model (2) reports the regression results with the inclusion of control variables. It is shown that Digital Literacy still exerts a significantly positive impact on rural residents' sports participation behavior (β = 0.257, < 0.001). An examination of the coefficients of the control variables reveals that gender, age, educational level, political status, marital status, and health status all have significant impacts. This result indicates that rural residents' sports participation behavior exhibits distinct heterogeneous characteristics. Models (3–5) present the regression results with the sequential inclusion of mediating variables. It is found that the regression coefficients of the three mediating variables are significantly positive, and this result remains consistent in the subsequent full-model regression.

**Table 2:** Benchmark regression results.

Variable	(1)	(2)	(3)	(4)	(5)	(6)
Digital Literacy	0.289^***^	0.257^***^	0.132^***^	0.172^***^	0.228^***^	0.0661^***^
	(0.0184)	(0.0191)	(0.0200)	(0.0207)	(0.0195)	(0.0215)
Gender	–	0.163^***^	0.0727^**^	0.190^***^	0.152^***^	0.111^***^
		(0.0295)	(0.0309)	(0.0319)	(0.0295)	(0.0327)
Age	–	−0.00801^***^	0.00431^***^	0.00665^***^	−0.00969^***^	0.0120^***^
		(0.00136)	(0.00143)	(0.00148)	(0.00138)	(0.00154)
Educational level	–	0.0567^***^	−0.0234^**^	−0.00835	0.0557^***^	−0.0572^***^
		(0.00956)	(0.0102)	(0.0104)	(0.00958)	(0.0108)
Political affiliation	–	0.952^***^	0.674^***^	0.669^***^	0.927^***^	0.483^***^
		(0.121)	(0.126)	(0.130)	(0.121)	(0.132)
Marital status	–	−0.626^***^	−0.508^***^	−0.572^***^	−0.636^***^	−0.499^***^
		(0.0378)	(0.0395)	(0.0408)	(0.0378)	(0.0417)
Health status	–	0.0643^***^	0.0438^***^	0.0435^***^	0.0866^***^	0.0526^***^
		(0.0145)	(0.0151)	(0.0157)	(0.0148)	(0.0162)
Digital usage behavior	–	–	1.431^***^			1.008^***^
			(0.0360)			(0.0391)
Human capital accumulation	–	–	–	2.425^***^		2.105^***^
	–	–	–	(0.0479)		(0.0497)
Level of social capital	–	–	–	–	0.116^***^	0.110^***^
	–	–	–	–	(0.0135)	(0.0148)
Constant	−1.082^***^	−0.555^***^	−0.870^***^	−1.493^***^	−1.310^***^	−2.316^***^
	(0.0738)	(0.103)	(0.108)	(0.114)	(0.136)	(0.151)
Pseudo R2	0.009	0.035	0.097	0.146	0.038	0.172
Observations	19815	19815	19815	19815	19815	19815

^**^p < 0.01,

^***^p < 0.001.

### 4.2 Robustness test

It is worth noting that rural residents who frequently participate in physical exercise may have higher Digital Literacy, leading to a certain counterfactual estimation issue. To avoid biased estimation results caused by sample selection bias, robustness tests were conducted on the regression results using the Heckman two-stage method and instrumental variable (*IV*) method. Models (1) and (2) report the regression results of the Heckman two-stage method. In the first stage, the probability of rural residents' sports participation behavior was estimated using a Logistic model to derive λ for each resident. In the second stage, this ratio (λ), Digital Literacy, and other control variables were introduced to estimate the impact of Digital Literacy on rural residents' sports participation behavior. It can be seen from the results that in the OLS regression results after incorporating λ, for each one-unit increase in rural residents' Digital Literacy, their sports participation behavior increases by 0.09 < 0.001), which is consistent with the benchmark regression results. Although different methods can mitigate sample self-selection bias as much as possible, endogeneity issues caused by reverse causality and omitted variable bias cannot be completely ruled out. Online shopping was selected as the instrumental variable, sourced from the CFPS questionnaire item: “Do you often shop online?” Responses of “Yes” were coded as 1, and “No” as 0, making it a dichotomous variable. First, the relevance of the instrumental variable: frequent online shopping by residents often implies strong digital technology usage ability, thereby enabling them to obtain more traffic information and achieve convenient consumption. Second, the exogeneity of the instrumental variable: no relevant evidence suggests that online shopping exerts an impact on rural residents' sports participation behavior through Digital Literacy. The two-stage least squares (2SLS) method was employed for estimation. It was found that the instrumental variable in the first stage is significant at the 1% level, indicating a high correlation between online shopping and Digital Literacy. Meanwhile, the *F*-statistic is greater than 10 (28.066), rejecting the null hypothesis that the instrumental variable is unidentifiable. In the second stage, the coefficient of the core explanatory variable remains significant at the 1% level. It can be concluded that online shopping is a valid instrumental variable, rejecting the null hypothesis of weak instrumental variables. Through the above robustness test analyses, the impact of Digital Literacy on rural residents' sports participation behavior is further verified.The verification results are shown in Table 3.

**Table 3:** Robustness test results.

Variable	(1) Phase one (logit)	(2) Stage two (ordinary least square model)	(3) Phase one	(4) Stage two
Digital Literacy	0.257^***^	0.0989^***^	–	0.369^***^
	(0.0189)	(0.0185)	–	(0.136)
Imr	–	0.228^**^	–	–
	–	(0.109)	–	–
Online shopping	–	–	0.0579^***^	–
	–	–	(0.011)	–
Kleibergen-Paap rk Wald F statistic	–	–	–	28.066
Control variables	Control	Control	Control	Control
Observations	19,815	19,815	19,815	19,815
R-squared	–	0.048	–	0.428

^**^p < 0.01,

^***^p < 0.001.

### 4.3 Testing for mediation effects

Based on the hypotheses derived earlier, digital usage behavior, human capital accumulation, and social capital level may play important mediating roles. Three mediating effect test methods were employed for verification. First, the stepwise regression method. Referring to the mediating effect test procedure proposed by Wen Zhonglin (50), it was found that all three mediating variables exert a significantly positive impact on rural residents' sports participation behavior. On the basis of the main effect, the mediating variables were sequentially incorporated into the regression, and the results remained unchanged, initially verifying the existence of the mediating effect. The Bootstrap method was employed to estimate the confidence intervals of the mediating effect, with 2,000 repeated samplings and a 95% confidence interval. It was found that the confidence intervals of all paths do not contain 0, indicating that the mediating effect hypothesis holds. Since the mediating effect analysis of the logistic model differs from that of the linear regression model, direct calculation of the mediating proportion through regression coefficients may lead to errors. Therefore, the KHB mediating test method was employed in this study to estimate the mediating proportion. It was found that the total proportion of indirect effects is 77.61%, among which digital usage behavior accounts for 42.64%, human capital accumulation for 44.59%, and social capital level for 9.91%. It can be concluded that regardless of the test method employed, Digital Literacy can promote rural residents' sports participation behavior through three pathways: stimulating digital usage behavior, facilitating human capital accumulation, and enhancing social capital level. The results are shown in Table 4.

**Table 4:** Testing for mediating effects.

Variable	(1)	(2)	(3)	(4)	(5)	(6)	(7)
	Sports participation behavior	Digital usage behavior	Sports participation behavior	Human capital accumulation	Sports participation behavior	Level of social capital	Sports participation behavior
Digital Literacy	0.0608^***^	0.0957^***^	0.0300^***^	0.0653^***^	0.0375^***^	0.266^***^	0.0535^***^
	(0.00446)	(0.00395)	(0.00434)	(0.00434)	(0.00421)	(0.00999)	(0.00453)
Digital usage behavior	–	–	0.322^***^	–	–	–	–
	–	–	(0.00764)	–	–	–	–
Human capital accumulation	–	–	–	–	0.357^***^	–	–
	–	–	–	–	(0.00680)	–	–
Level of social capital	–	–	–	–	–	–	0.0276^***^
	–	–	–	–	–	–	(0.00314)
Control variables	Control	Control	Control	Control	Control	Control	Control
Constant	0.348^***^	0.238^***^	0.272^***^	0.539^***^	0.156^***^	6.477^***^	0.170^***^
	(0.0243)	(0.0214)	(0.0233)	(0.0236)	(0.0230)	(0.0543)	(0.0316)
Observations	19815	19815	19815	19815	19815	19815	19815
R-squared	0.054	0.195	0.131	0.106	0.168	0.078	0.058
Path	–	Lower limit (95% CI)	–	–	–	Upper limit (95% CI)	–
Digital usage behavior							
Direct effect	–	0.0293	–	–	–	0.0349	–
Indirect effects	–	0.0208	–	–	–	0.0380	–
Human capital accumulation							
Direct effect	–	0.0219	–	–	–	0.0286	–
Indirect effects	–	0.0282	–	–	–	0.0443	–
Level of social capital							
Direct effect	–	0.005	–	–	–	0.009	–
Indirect effects	–	0.045	–	–	–	0.063	–
KHB decomposition (total effect)	–	–	–	0.295^***^(Z = 14.07)	–	–	–
Direct effect	–	–	–	0.066 ^**^(Z = 14.07)	–	–	–
Indirect effects	–	–	–	0.228^***^(Z=21.89)	–	–	–
Indirect effect proportion/%	–	–	–	77.61%	–	–	–
Contribution rate of each	–	Digital Usage Behavior	–	Human capital accumulation	–	Level of social capital	–
	–	42.64%	–	44.59%	–	9.91%	–

^**^p < 0.01,

^***^p < 0.001.

### 4.4 Heterogeneity test

During the process of digital transformation, China is facing the challenge of imbalance and inadequacy in the development of digital technologies between urban and rural areas, while the integration of advanced technologies such as information networks, artificial intelligence, big data, and cloud computing with modern rural development is being rapidly advanced. Numerous studies have also demonstrated that there are significant demographic characteristic differences in sports participation among rural residents (51, 52). We further estimate the heterogeneous effects of digital literacy on sports participation among rural residents. From the perspective of gender, digital literacy has a significant impact on sports participation among rural residents of different genders, and comparative regression coefficients reveal that this impact is more pronounced for males. To distinguish whether there are differences in exercise participation across different age groups, a grouped regression analysis was conducted based on the age classification set forth by the World Health Organization (WHO), which defines youth (18–44 years), middle-aged (45–59 years), and elderly (60 years and older). As shown in Table 5, The findings reveal that digital literacy has a greater influence on sports participation among middle-aged rural residents. From the marital perspective, rural residents who are married exhibit higher levels of digital literacy compared to their unmarried counterparts and are more inclined to participate in sports activities.

**Table 5:** Tests for heterogeneity by gender, age and marital status.

Variable	(1)	(2)	(3)	(4)	(5)	(6)	(7)
	Female	Male	Young	Middle-aged	Elderly	Unmarried	Married
Digital literacy	0.241^***^	0.282^***^	0.253^***^	0.304^***^	0.212^***^	0.183^***^	0.289^***^
	(0.0274)	(0.0268)	(0.0249)	(0.0350)	(0.0621)	(0.0403)	(0.0217)
Constant	−0.771^***^	−0.189	−0.686^***^	−1.913^***^	−1.285^***^	0.214	−1.624^***^
	(0.148)	(0.141)	(0.119)	(0.198)	(0.337)	(0.210)	(0.109)
Pseudo R2	0.027	0.044	0.040	0.016	0.013	0.032	0.013
Observations	9,949	9,866	13,313	5,091	1,410	5,394	14,421

^**^p < 0.01,

^***^p < 0.001.

## 5 Discussion

Entering the era of digital economy, the problem of unimpeded urban information flow and blocked rural information flow caused by unequal access to and use of the Internet has become increasingly prominent. The digital divide is gradually shifting from the first-level divide characterized by “access disparity” to the second-level and third-level divides represented by “usage disparity” and “benefit disparity” (53). Against this backdrop, although a growing number of studies have adopted digital technology as an effective means to intervene in physical activities of different populations (54, 55), it is easily overlooked that the evaluation and verification of the effectiveness of physical activities based solely on the single dimension of whether the Internet is used still lack certain rigor and reliability. Furthermore, an empirical analysis framework for the rural resident group has not yet been established, and the gap in physical exercise effectiveness caused by different levels of Digital Literacy requires attention and resolution. Based on this, this study utilized large-scale comprehensive survey data to establish a framework model between rural residents' Digital Literacy and sports participation behavior, analyzed and tested the internal transmission paths therein, and further explored the existing heterogeneous performance of demographic characteristics. In this study, the two-stage test, instrumental variable method, and various mediating test methods were also employed to conduct robust estimation of the results, supporting the reliability of the results.

The main results of this study confirm that Digital Literacy exerts a significant promoting effect on rural residents' sports participation behavior, and this finding is generally consistent with previous studies (56). Owning electronic devices such as mobile phones and computers does not mean being able to use these tools proficiently. On the one hand, emerging technological products such as intelligent electronic devices have numerous functions and complex operational processes, requiring a certain amount of time to read instructions or participate in skill training to master; insufficient experience hinders rural residents from applying them well in daily life. On the other hand, rural residents with insufficient or low levels of Digital Literacy only engage in addictive behaviors through simple operations such as liking, commenting, or reposting, rather than shifting to actual exercise participation behaviors. Meanwhile, it may also lead to the leakage of residents' safety and privacy or participation in fraudulent fitness course marketing and consumption. Existing studies have shown that Digital Literacy can enhance various indicators such as physical health and mental health of individuals (57). For example, residents with high Digital Literacy are motivated to further utilize digital health resources, thereby promoting more active digital health behaviors (58). It is worth noting that the current study is limited to rural residents in China. This is because China is gradually strengthening the implementation of policies such as Digital China, digital rural construction, and the rural revitalization strategy; improving Digital Literacy may further enhance the exercise level of rural residents, which has certain practical value and policy implications. However, due to the unbalanced characteristics of the digital divide across countries worldwide, factors such as geographical location, network environment, and economic conditions limit the access rate and usage rate of devices among rural residents. For instance, network resources and infrastructure in the United States and South Africa remain underdeveloped, residents lack solid Internet operation skills and digital thinking, and access to healthcare services is still insufficient (59). This is also prevalent in other countries such as Italy, Portugal, and Spain (60). Improving Digital Literacy can help them obtain valuable health information, access advanced medical information, and guide positive and healthy exercise behaviors (61). Therefore, ensuring the coverage of digital technology infrastructure is only one-sided; it is also necessary to focus on addressing the problems of rural residents' insufficient basic knowledge of digital technology and difficulties in practical application. This is particularly important for the elderly digital vulnerable group, as it can help them better promote exercise participation and alleviate health inequality.

This study also found that digital usage behavior, human capital accumulation, and social capital level are found to play a crucial mediating role in the impact of Digital Literacy on rural residents' sports participation behavior. With the continuous improvement of digital infrastructure in rural areas, the digital poverty situation in rural China has been alleviated, and the popularity of accessible electronic devices and online information platforms among rural residents has been increasing. Cetindamar et al. developed a theoretical analysis framework of “Theory of Planned Behavior (TPB) + Digital Literacy” and empirically verified that Digital Literacy is an effective supplement to the TPB model (62). Specifically, the improvement of rural residents' technical capabilities and the transformation of their traditional concepts enable them to better accept new technologies, utilize digital resources to meet diverse needs such as social interaction, entertainment, and leisure, and further enhance the breadth and depth of their digital technology usage. For example, a South Korean survey study showed that elderly individuals who participated in Digital Literacy education programs exhibited a significant improvement in their ability to use smartphones and record videos, thereby facilitating continuous health management (63). The implementation of the Broadband China Strategy has increased the Internet coverage rate in rural areas. Studies have shown that individuals can utilize the Internet as an information transmission medium for social communication and lifelong learning, acquire sports health information, and thereby internalize it into sports participation behavior (64).

Human capital accumulation is another crucial mediating mechanism. Our study has confirmed that human capital accumulation exerts a positive mediating effect between Digital Literacy and rural residents‘ sports participation behavior, which has received little attention in existing studies. Human capital refers to the accumulation of knowledge and creativity, and its importance in the process of digital transformation has become increasingly prominent. Individuals with high Digital Literacy can continuously enhance their digital usage skills through online learning platforms, including information retrieval, needs assessment, and program monitoring (65), and can better utilize big data algorithms to develop scientific fitness plans. In contrast, rural residents with low Digital Literacy may lack sufficient understanding and cognition of device interface operations, functional permissions, or intelligent services, and thus be unable to use digital technology for health management or receive remote fitness guidance. Meanwhile, they may also receive negative and false promotions from various merchant marketing accounts, make incorrect exercise decisions, and exhibit a psychological perception of “being able to see but unable to use” regarding Internet technology. Therefore, in the process of fostering rural residents' Digital Literacy, attention should be paid to individual endowment differences in the use of digital technology. Systematic education and training should be utilized to form and strengthen digital awareness and digital concepts, so as to better facilitate their participation in fitness activities.

The social capital accumulation effect of Digital Literacy is a crucial factor in enhancing rural residents' sports participation behavior. Studies conducted in five rural and urban areas of New South Wales, Australia, have found that rural residents exhibit a higher level of participation in community organizations, neighborhood connections, and voluntary community activities (66). The improvement of Digital Literacy facilitates rural residents' ability to acquire, manage, and exchange information and knowledge, promotes the accumulation of individual social relationship resources, and thereby fosters the accumulation of social capital (67). The content of social relations in rural China is gradually shifting to digital platforms, breaking geographical and temporal constraints through intelligent integration, and bringing opportunities for the improvement of residents' social capital stock. Specifically, the higher the level of Digital Literacy of residents, the more proficient they are in using digital technology to establish and maintain social relations, promote the mutual flow of information resources (68), and form stable sports interest-based communities. The development of social capital effectively mobilizes individuals' ability to participate in physical activities. For example, actors with abundant online social capital are embedded with a greater capacity for information resources (69) and can achieve sports participation through the following three pathways: First, instant messaging platforms. Real-time online video, text, and voice communication creates opportunities for connection to facilitate rural residents' sports sharing and discussions. Second, cutting-edge technologies such as virtual reality (VR) platforms, cloud data, and other VR technologies have been introduced into rural communities, enhancing the sense of presence and novelty of residents' digital sports experiences. Third, smart sports social platforms. The Internet expands the weak-tie networks of residents with high Digital Literacy, providing more expressive interaction opportunities, such as online event viewing, product and service consumption, and exercise behavior incentives.

Results from the heterogeneity analysis revealed that Digital Literacy exerts a more pronounced promoting effect on the sports participation behavior of male, middle-aged, and married rural residents. From the gender perspective, in the process of rural modernization, rural men tend to focus more on agricultural market trends, policy support, and technical knowledge. In contrast, rural women are often at a disadvantage in accessing information due to cultural customs or family responsibilities. From the age group perspective, young and middle-aged rural residents are more receptive to new things, more proficient in mastering digital technologies, and have higher levels of Digital Literacy. From the marital status perspective, married rural residents have the family economic conditions to jointly share the cost of purchasing digital technology equipment with their spouses, are willing to try and explore more additional functions of the equipment, and thus provide support for sports participation.

This study has several limitations. First, we employed large-scale comprehensive survey data to infer the causal relationship between Digital Literacy and rural residents‘ sports participation behavior; however, this study is mostly based on cross-sectional research. In the future, we will consider methods such as quasi-natural experiments and randomized controlled trials to provide more detailed and in-depth empirical analyses. Second, the selected data was sourced solely from rural areas in China, resulting in a lack of external validity and generalizability of the research findings. In the future, we will consider comprehensive comparisons across Europe, Africa, and other regions worldwide to provide a reference for improving global Digital Literacy levels. Finally, both Digital Literacy and sports participation behavior were measured through self-reports. Regarding the Digital Literacy indicator, the differences in residents' digital technology tool systems and their actual usage have not been fully considered. In subsequent research, objective measurement methods will be adopted to enhance the rigor of indicator selection. For the sports participation behavior indicator, measuring only exercise frequency and duration is insufficiently specific. It is further necessary to incorporate exercise intensity, types, and organizations into the scope of reference to ensure the representativeness and accuracy of the research findings.

## 6 Conclusions

Based on the findings of this study, the conclusions drawn are as follows:

Although this study has certain limitations, it has also made supportive contributions and derived several valuable research conclusions. First, it empirically examined the impact and mechanisms of Digital Literacy on rural residents' sports participation behavior, addressed the limitation of using a single dimension of Internet usage in previous studies, and provided empirical references and policy implications for better enhancing rural residents' exercise levels and improving their health status. Second, it uncovered the potential mechanisms of action of the study: the improvement of rural residents' Digital Literacy can enhance their digital usage behavior, facilitate social learning and skill training, and establish reciprocal and trusting social network relationships. These influence pathways can better guide rural residents to actively participate in various sports activities. Third, the instrumental variable method and two-stage test were applied to the regression model, enhancing the overall robustness of the results. Furthermore, the heterogeneity analysis revealed that improving the Digital Literacy level of male, middle-aged, and married rural residents tends to result in more active exercise participation, which provides valuable information for formulating personalized policy plans in subsequent research.

## Data Availability

The original contributions presented in the study are included in the article/supplementary material, further inquiries can be directed to the corresponding author.
