# Supplementary material for: The impact of digital literacy on rural residents' sports participation behavior:evidence from China
Source: Front Public Health. 2026 Apr 9;14:1781770. doi: 10.3389/fpubh.2026.1781770 (PMC13102839; doi:10.3389/fpubh.2026.1781770)
Supplement: Supplementary file 1 [file Supplementary_File_1.docx]

**Supplementary Material 1**

**1 Complete Wording of Survey Questions and Coding Rules for the Dependent Variable**

Dependent Variable: Sports Participation Behavior of rural residents

1.1 Core Survey Items (CFPS Individual Questionnaire, "Health and Lifestyle" Module)

(1) How often do you participate in physical therapy? How often do you engage in physical and leisure activities? (2) Generally speaking, how many minutes do you usually exercise each time you participate in physical and leisure activities?

1.2 Coding Rules

For the first item, the original answers and initial coding are as follows: Never participated = 1, Less than once a month on average = 2, Multiple times a month on average = 3, 1~2 times a week on average = 4, 3~4 times a week on average = 5, 5 or more times a week on average = 6, Once a day = 7, More than twice a day = 8.

For the second item, the original answers are continuous values (Unit: minutes), which were coded using binary variables: Each exercise ≥ 30 minutes = 1, and each exercise < 30 minutes = 0.

Final coding rules for the dependent variable (Sports Participation Behavior): "An average weekly exercise frequency ≥ 3 times and each exercise duration ≥ 30 min" is defined as participating in physical exercise, assigned a value of 1; otherwise, it is assigned a value of 0.

**2 Complete Wording of Survey Questions and Coding Rules for the Independent Variable**

Independent Variable: Digital Literacy of rural residents

2.1 Core Survey Items

Individuals' functional cognition of Internet technology was used to reflect their Digital Literacy level. The survey design included 5 items to measure the perceived importance of the Internet in different life domains, with specific sub-items as follows: Online Digital Work Literacy (the importance of the Internet for work), Digital Entertainment Literacy (the importance of the Internet for leisure and entertainment), Digital Social Literacy (the importance of the Internet for maintaining contact with family and friends), Digital Learning Literacy (the importance of the Internet for learning), and Digital Life Literacy (the importance of the Internet for daily life).

2.2 Coding Rules

Each sub-item was coded using a 5-point Likert scale: Very unimportant = 1, Unimportant = 2, Neutral = 3, Important = 4, Very important = 5. Exploratory Factor Analysis (EFA) was performed on the 5 items; the KMO value was 0.788, and Cronbachαwas 0.727, which met the statistical requirements for factor analysis. After rotation, a common factor (Digital Literacy factor) was extracted, with a cumulative variance contribution rate of 66.4% and a factor loading coefficient range of 0.491 to 0.648. The factor score of each sample was used as the composite index of Digital Literacy, with a value range of 1~5 (the higher the score, the higher the level of Digital Literacy).

**3 Complete Wording of Survey Questions and Coding Rules for Mediating Variables**

3.1 Digital Usage Behaviour

3.1.1 Core Survey Items

(1) Do you use mobile Internet (e.g., mobile phones, tablets)? (Yes/No) (2) Do you use computer Internet (e.g., desktop computers, laptops)? (Yes/No)

3.1.2 Coding Rules

Each item was coded using a binary variable: Yes = 1, No = 0. The composite index of Digital Usage Behaviour is a binary variable: if the respondent used mobile Internet or computer Internet (i.e., the sum of the two items ≥ 1), it was assigned a value of 1; if neither was used, it was assigned a value of 0.

3.2 Human capital accumulation

3.2.1 Core Survey Items

(1) Have you participated in learning activities (including offline and online learning) in the past 12 months? (Yes/No) (2) How many times do you use the Internet for learning activities on average per month? (Specific value/No online learning)

3.2.2 Coding Rules

For the first item: Yes = 1, No = 0; for the second item: it was converted into a binary variable based on the presence of online learning behavior (with online learning behavior = 1, without online learning behavior = 0). The composite index of Human capital accumulation is a binary variable: the average value of these two items was calculated; if the average value ≥ 0.5 (i.e., with learning behavior and/or online learning behavior), it was assigned a value of 1; if the average value < 0.5, it was assigned a value of 0.

3.3 Level of social capital

3.3.1 Core Survey Items

(1) How much do you trust your parents? (Parental Trust) (2) How much do you trust your neighbors? (Neighbor Trust) (3) How do you evaluate your interpersonal relationships (popularity) in the local area? (Popularity)

3.3.2 Coding Rule

Each item was coded using a 10-point scale: 0 = Complete distrust/Very poor, 1~9 = Neutral, 10 = Complete trust/Very good. The composite index of Level of social capital is the sum of the three item scores, with a score range of 0~10 (the higher the score, the higher the Level of social capital).

**4 Complete Wording of Survey Items and Coding Rules for Key Control Variables**

Control variables were selected from the "Basic Personal Information" and "Health Status" modules of the CFPS Individual Questionnaire, and the complete survey items and coding rules are as follows:

Gender: Survey Item: What is your gender? Coding: Female = 0, Male = 1.

Age: Survey Question: What is your date of birth? Coding: The actual age was calculated as (survey year - birth year), with a value range of 18~100 years old (only adult samples were included in this study).

Educational Level: Survey Question: What is your highest educational achievement? Coding: Primary school and below = 0, Junior high school = 1, Senior high school (general high school, secondary vocational school, technical school) = 2, Junior college = 3, Bachelor's degree and above = 4.

Political Status: Survey Item: What is your political status? Coding: Masses = 0, Communist Party of China (CPC) member = 1.

Marital Status: Survey Item: What is your current marital status? Coding: Unmarried (including single, divorced, widowed) = 0, Married (including married and cohabiting) = 1.

Health Status: Survey Question: How do you evaluate your current physical health status? Coding: Unhealthy = 1, Average = 2, Relatively healthy = 3, Very healthy = 4, Extremely healthy = 5.
